# The Impact of BMI on Perioperative Urine Volume and Renal Function in Living Donor Kidney Transplantation

**DOI:** 10.7759/cureus.103584

**Published:** 2026-02-14

**Authors:** Masahiro Todaka, Tadasuke Ando, Hiroyuki Fujinami, Naoyuki Yamanaka, Shinro Hata, Toru Inoue, Toshitaka Shin

**Affiliations:** 1 Urology, Faculty of Medicine, Oita University, Yufu, JPN; 2 Organ Transplantation Promotion Project, Faculty of Medicine, Oita University, Yufu, JPN

**Keywords:** body mass index, living donor kidney transplantation, obesity, renal function, urine volume

## Abstract

Introduction: Obesity is a well-recognized risk factor for various systemic disorders and has also been shown to influence perioperative outcomes in surgical fields. In the context of living donor kidney transplantation (LDKT), increased BMI has been associated with greater technical complexity and a higher incidence of postoperative complications, including wound infection and delayed graft function (DGF). We explored the impact of BMI on the perioperative urine volume (UV) and renal function in LDKT.

Methods: We conducted a retrospective analysis at a single institution to assess the impact of BMI on outcomes following LDKT. Patients were stratified according to BMI into the obese group (Og) (BMI ≥ 25 kg/m²) and the non-obese group (nOg) (BMI < 25 kg/m²). Between April 2010 and December 2024, 118 patients underwent LDKT at Oita University Hospital. After excluding patients who experienced acute rejection, underwent repeat kidney transplantation, or developed vesicoureteral anastomotic complications, 109 patients remained eligible for analysis. We compared outcomes and UV within 14 days postoperatively, and renal function between the Og and nOg. The mean (SD) postoperative follow-up period was 79.7(47.2) months in the nOg and 56.1 months (47.5) in the Og (p = 0.0156).

Results: A total of 109 patients were analyzed, comprising 36 patients in the Og and 73 in the nOg. Baseline demographic and clinical characteristics were generally comparable between the two groups, including age, sex, comorbidity, donor BMI, duration from dialysis initiation to transplantation, immunosuppressive regimens. However, baseline serum creatinine levels were significantly higher in obese recipients than in non-obese recipients (p = 0.005). Postoperatively, UV on postoperative day 1 (POD 1) was significantly lower in the Og (p = 0.02). Furthermore, the mean rate of creatinine reduction and the mean estimated glomerular filtration rate (eGFR) increase were significantly higher in the nOg from POD1 to POD4 (p<0.05). No significant differences were observed with respect to warm ischemia time (WIT), total ischemia time (TIT), length of hospital stay, or the incidence of postoperative complications. Multivariate analysis identified obesity as an independent predictor of reduced UV on POD1 (p = 0.00978).

Conclusion: Obese recipients undergoing LDKT demonstrated significantly lower UV on POD1 and a slower initial recovery of graft function. However, these early physiological differences did not translate into a higher incidence of major postoperative complications or prolonged length of hospital stay. These findings suggest that although early UV may be lower in obese recipients, careful and individualized perioperative fluid management is essential to maintain adequate graft perfusion.

## Introduction

In Japan, the prevalence of obesity (BMI ≥ 25kg/m^2^) has increased in recent years, rising from 24.3% to 26.3%. There has been a particularly marked increase among men, rising from 29.3% to 31.8%[1]. Among patients with end-stage kidney disease in Japan, obesity prevalence increased significantly between 2006 and 2019, with annual growth rates of 3.36% and 2.86% for men and women, respectively [2]. Adipose tissue is not only an energy storage site but also a major endocrine gland that secretes adipocyte-derived cytokines [3]. Therefore, obesity can cause health problems, such as hypertension, stroke, and ischemic heart failure, and in the field of living donor kidney transplantation (LDKT), obesity is associated with increased rates of delayed graft function (DGF), wound-related complications, and surgical site infection (SSI) [4,5]. Although previous studies have extensively evaluated the impact of obesity on graft outcomes and postoperative complications in kidney transplant recipients, perioperative urine volume (UV) and its implications for early postoperative management in LDKT have not been sufficiently investigated. Perioperative fluid management is a critical component of LDKT, particularly during the immediate postoperative period when UV is commonly used as a surrogate marker of graft perfusion and renal function. UV plays a central role in the early diagnosis of acute kidney injury (AKI) and in guiding fluid administration strategies [6]. However, UV is influenced not only by renal perfusion but also by patient-specific factors, such as body composition and fluid distribution, raising concerns regarding its interpretation in obese recipients [7]. Therefore, this study compared perioperative outcomes, including UV, between the obese group (Og) and the non-obese group (nOg). Limited data are available in the field of LDKT regarding the relationship between perioperative UV, fluid distribution, and early graft perfusion and renal function. Furthermore, the influence of recipient BMI on early postoperative UV and its clinical implications has not been sufficiently investigated. Therefore, we conducted this study to further elucidate these associations.

## Materials and methods

Study design 

**Figure 1:**
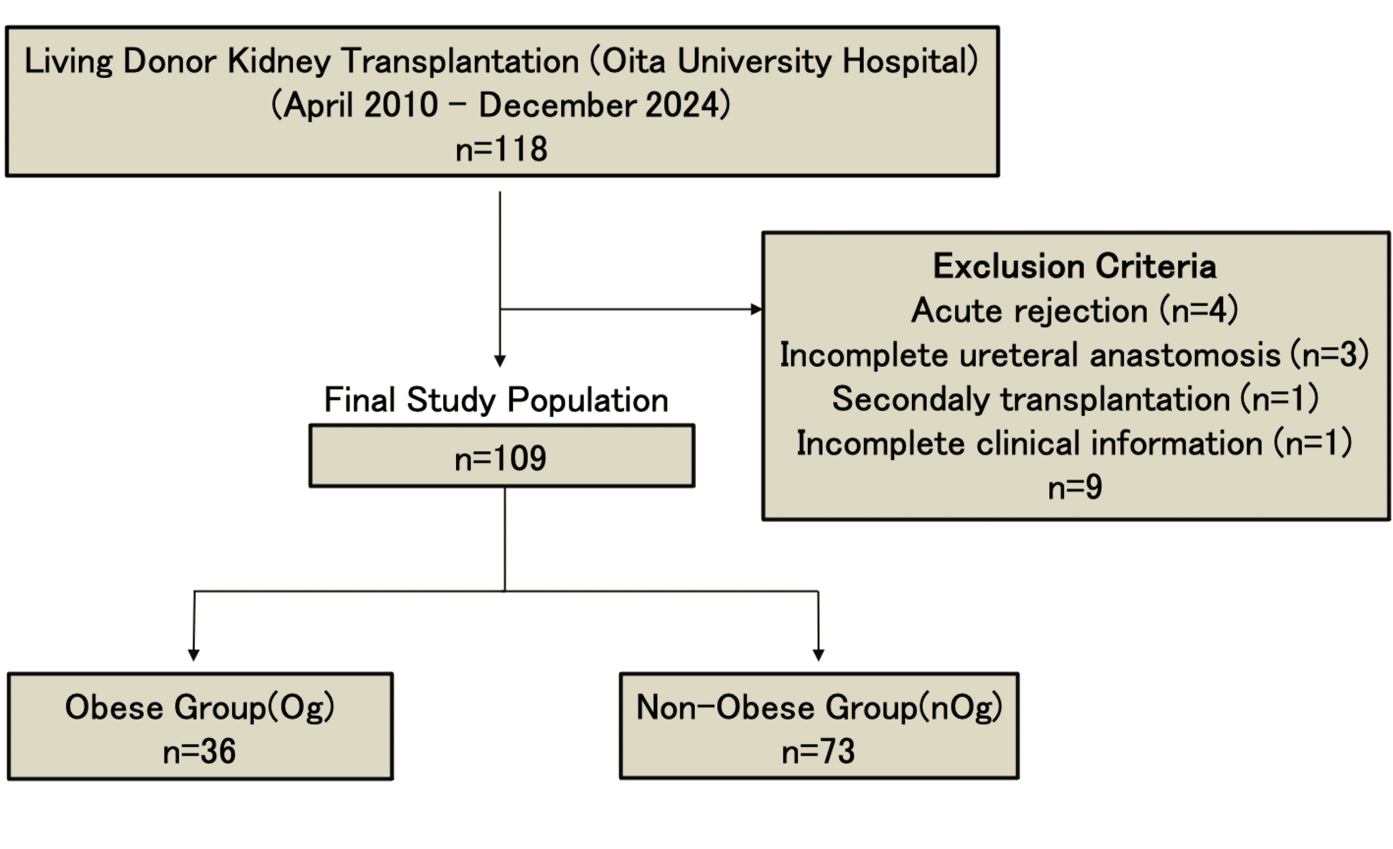
Patient selection flow chart Og: Obese group; nOg: Non-obese group

Patients selection flow chart is shown in Figure 1. We conducted a retrospective observational analysis at a single institution to evaluate the relationship between obesity and perioperative clinical parameters in patients undergoing LDKT at Oita University between April 1, 2010, and December 31, 2024 by comparing obesity and non-obesity with respect to UV, transplanted kidney function, and postoperative complications. Postoperative complications were evaluated using the Clavien-Dindo classification (C-D classification) [8]. The study was conducted in accordance with the Declaration of Helsinki and good clinical practice guidelines. It was also approved by the Ethics Committee of Oita University Hospital (study number: 2559).

Participants

Eligible patients were ≥ 20 years of age, who underwent LDKT at Oita University between April 1, 2010, and December 31, 2024 and submitted written informed consent from the subjects (or their guardians) before enrolling in the study. Patients with acute rejection, secondary kidney transplantation, and vesicoureteral anastomosis failure were excluded. Patient data (age, sex, preoperative donor and recipient BMI prior to LDKT, duration of hemodialysis (HD), medical history, 24-hour UV, 24-hour fluid intake, serum creatinine, eGFR, perioperative complications, warm ischemic time (WIT), and total ischemic time (TIT)) were collected from medical records. Blood sample was collected prior to the morning immunosuppressant dose and breakfast. Recipients were categorized according to BMI, with a threshold of 25 kg/m² used to define obesity the group of patients with obesity as the Og and the group of patients without obesity as the nOg. This definition of obesity was in accordance with World Health Organization (WHO) recommendations for Asian populations. The backgrounds and data of the Og and nOg were compared.

Statistical analysis

Continuous variables were compared with the Mann-Whitney U test and the Student’s t-test. Univariate and multivariate analysis of categorical variables were performed using single regression and stepwise multiple regression analysis to adjust for the appropriate number of clinically important confounders. Statistical analyses were performed using EZR (Saitama Medical Center, Jichi Medical University), a graphical user interface for R (The R Foundation for Statistical Computing, Austria), version 4.5.0, which is widely used for biostatistical analyses in clinical research. [9]. The statistical significance level was set at p < 0.05.

## Results

**Figure 2:**
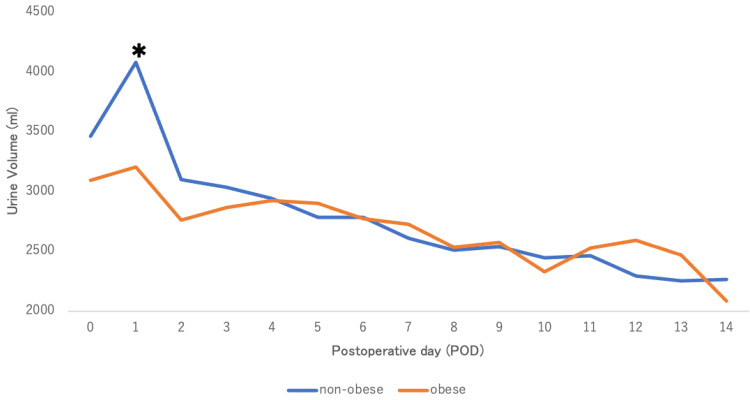
Perioperative UV An asterisk (*) indicates a statistically significant difference as determined by the t-test (p < 0.05). UV: Urine volume

**Figure 3:**
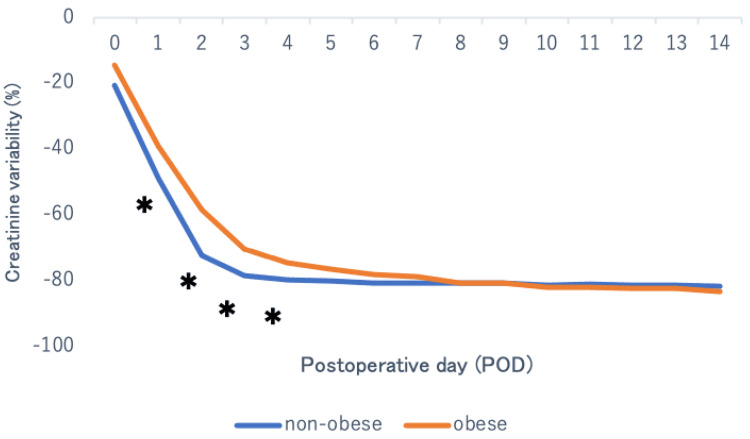
Postoperative variability in serum creatinine An asterisk (*) indicates a statistically significant difference as determined by the t-test (p < 0.05).

**Figure 4:**
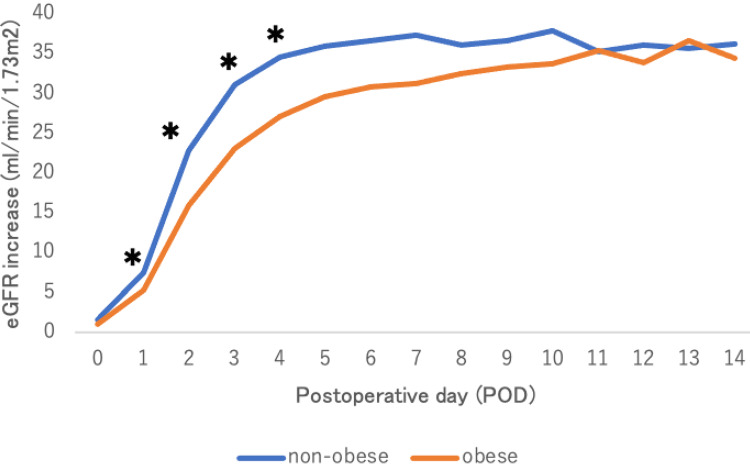
Postoperative increase in eGFR An asterisk (*) indicates a statistically significant difference as determined by the t-test (p < 0.05). eGFR: Estimated glomerular filtration rate

Baseline variable characteristics are shown in Table 1. This study included 84 (77.1%) men and 25 (22.9%) women with a mean age (SD) of 49.6 (13.8) years. The mean BMI was 28.2 (1.9) kg/m² in the Og and 21.3 (1.9) kg/m² in the nOg. Donor BMI did not differ between the groups. Preoperative serum creatinine was significantly higher in the Og than in the nOg (p = 0.026), whereas preoperative eGFR was comparable between the groups. Baseline categorical characteristics are summarized in Table 2. The most common primary disease leading to chronic kidney disease is diabetic nephropathy (n=28, 25.7%). The cases of pre-emptive kidney transplants (PEKT) were 26 (23.9%). The regimen of immunosuppressant was cyclosporine (CyA), mycophenolate mofetil (MMF), methylprednisolone (MP), and basiliximab in only one patient, and tacrolimus in all others. Blood-type-incompatible kidney transplants were performed in 45 cases (41.3%). All patients received rituximab one month before transplantation and underwent multiple plasma exchanges immediately before transplantation. Variable perioperative outcomes are shown in Table 3. There were no significant differences between the two groups in intraoperative WIT, TIT, and total perioperative fluid and blood transfusion volumes. Categorical perioperative outcome is shown in Table 4. No C-D classification grade ≥III complications occurred in the Og, whereas five cases were observed in the nOg, with no statistically significant difference. Postoperative admission period was also comparable between groups. As Figure 2 shows, in univariate analysis, mean UV on postoperative day 1 (POD 1) was significantly lower in the Og compared with the nOg . The reduction in postoperative serum creatinine was significantly greater in the nOg from PODs 1-4 in Figure 3. Furthermore, the postoperative increase in estimated glomerular filtration rate (eGFR) was significantly greater in the nOg from PODs 1-4 in Figure 4.

**Table 1:** Baseline variable characteristics of recipients and donors Data is presented as mean ± SD. An asterisk (*) indicates a statistically significant difference as determined by the t-test (p < 0.05). eGFR: Estimated glomerular filtration rate

Variable	Overall (n=109)	Non-obese (n=73)	Obese (n=36)	p value
Age, years	49.6 ± 13.8	51.1 ± 14.2	46.6 ± 12.6	0.104
BMI, kg/m²	23.6 ± 3.78	21.3 ± 1.9	28.2 ± 1.9	<0.001*
Creatinine, mg/dL	9.44 ± 3.35	8.77 ± 3.0	10.8 ± 3.7	0.026*
eGFR, mL/min/1.73m²	5.90 ± 2.44	6.18 ± 2.5	5.30 ± 2.2	0.075
Dialysis period, days	1139.3 ± 1375	1313.4 ± 1584	821 ± 805	0.122
Donor age	61.0 ± 8.28	61.0 ± 8.7	61.0 ± 7.6	0.981
Donor BMI, kg/m²	23.5 ± 3.36	23.1 ± 3.4	24.3 ± 3.1	0.083
Donor creatinine, mg/dL	0.69 ± 0.17	0.68 ± 0.17	0.71 ± 0.16	0.403
Donor eGFR, mL/min/1.73m²	76.1 ± 16.9	75.6 ± 18.2	77.0 ± 14.1	0.681

**Table 2:** Baseline categorical characteristics of recipients Data is presented as n (%). HD: Hemodialysis; PD: Peritoneal dialysis; CyA: Cyclosporin; MMF: Mycophenolate mofetil; MP: Methylprednisolone; Tac: Tacrolimus; Basi: Basiliximab; Rit: Rituximab

Variable	Overall (n=109)	Non-obese (n=73)	Obese (n=36)	p value
Sex				0.148
Male	84 (77.1)	53 (72.6)	31 (86.1)	
Female	25 (22.9)	20 (27.4)	5 (13.9)	
Primary disease				0.474
Diabetic nephropathy	28 (25.7)	16 (21.9)	12 (33.3)	
Chronic glomerulonephritis	13 (11.9)	7 (9.6)	6 (16.7)	
Nephrosclerosis	11 (10.1)	7 (9.6)	4 (11.1)	
Others	57 (52.3)	43 (58.9)	14 (38.9)	
Comorbidity				
Hypertension	89 (81.7)	59 (80.8)	30 (83.3)	1.000
Dyslipidemia	44 (40.4)	26 (35.6)	18 (50.0)	0.213
Preemptive kidney transplantation	26 (23.9)	19 (26.0)	7 (19.4)	0.486
Dialysis				0.841
HD	65 (59.6)	42 (57.5)	23 (63.9)	
PD	2 (1.8)	1 (1.4)	1 (2.8)	
Transition from PD to HD	11 (10.1)	8 (11.0)	3 (8.3)	
Combination of PD and HD	5 (4.6)	3 (4.1)	2 (5.6)	
Immunosuppressant				0.478
CyA + MMF + MP + Basi	1 (0.9)	0 (0)	1 (2.8)	
Tac + MMF + MP + Basi	63 (57.8)	42 (57.5)	21 (58.3)	
Tac + MMF + MP + Basi + Rit	45 (41.3)	31 (42.5)	14 (38.9)	

**Table 3:** Variable perioperative outcomes Data is presented as mean ± SD. TIT: Total ischemic time; WIT: Warm ischemic time

Variable	Overall (n=109)	Non-obese (n=73)	Obese (n=36)	p value
TIT, min	167.4 ± 52.9	164 ± 55	174 ± 47	0.353
WIT, sec	231.4 ± 103.4	223 ± 69	250 ± 152	0.205
Infusion volume, mL,	6204.9 ± 1496.1	6326 ± 1518	5959 ± 1440	0.230
Postoperative hospital stay, days	19.3 ± 6.6	19.8 ± 7.4	18.4 ± 4.1	0.294

**Table 4:** Categorical perioperative outcome Data is presented as n (%). Details of C-D grade ≥ III complications included anastomotic stenosis (n=1), lymphocele (n=2), SSI (n=1), and diverticular perforation (n=1). C-D: Clavien-Dindo; SSI: Surgical site infection

Variable	Overall (n=109)	Non-obese (n=73)	Obese (n=36)	p value
Complication (≥C-D III)	5 (4.6)	5 (6.8)	0 (0)	0.169

A multivariate analysis using a generalised linear model is shown in Table 5, which identified obesity as an independent predictor of lower UV on POD 1 (R² = 0.150, p < 0.05).

**Table 5:** Multivariate regression analysis for POD 1 and POD 2 An asterisk (*) indicates a statistically significant difference as determined by the multivariable regression analysis (p < 0.05). TIT: Total ischemic time; WIT: Warm ischemic time; POD: Postoperative day

Variable	POD 1 p value	POD 2 p value
BMI (≥25 vs <25)	<0.05*	0.087
Donor BMI (≥25 vs <25)	0.604	0.474
Age (≥65 vs <65)	0.790	0.868
TIT	0.087	0.537
WIT	0.108	0.748
Dyslipidemia	0.106	0.272
Sex	0.596	0.800
Creatinine	0.699	0.844
R²	0.150	0.070

## Discussion

In this study, recipients in the Og exhibited significantly lower UV on POD 1 and a slower initial recovery of renal function compared with the nOg. Despite these early physiological differences, no significant differences were observed in C-D classification grade III or higher complications or length of hospital stay. These findings indicate that obesity influences early postoperative graft physiology without adversely affecting short-term clinical outcomes.

One possible explanation for the lower early UV observed in the Og is the altered endocrine environment associated with excess adipose tissue. Adipose tissue plays an active endocrine role, and excessive fat accumulation alters the secretion profile of adipocytokines, including reduced adiponectin and elevated leptin levels [10,11]. Adiponectin exerts anti-inflammatory and anti-atherosclerotic effects, and its receptors are expressed in glomeruli [12,13]. Previous experimental and clinical studies have suggested that decreased adiponectin activity is linked to podocyte dysfunction, increased urinary albumin excretion, and progression of renal injury [14]. In contrast, leptin enhances renal sodium reabsorption via sympathetic stimulation, increases blood pressure, and promotes proliferation of renal endothelial and mesangial cells, contributing to kidney injury [15]. It is plausible that multiple factors, including inflammatory markers, may have interacted in a complex manner, thereby contributing to the observed reduction in early postoperative UV in the Og [16].

Another plausible mechanism relates to altered fluid dynamics in obese patients. Obese patients may have an expanded extracellular fluid distribution, which could influence intravascular volume dynamics during the immediate postoperative period. To date, there are no published studies that have systematically examined third-space expansion and perioperative fluid management in obese patients, either in LDKT or in other surgical settings. Although direct evidence in kidney transplantation is limited, insights from critical care medicine provide useful perspectives. In the field of critical care, fluid resuscitation during the early phase of burn management based on actual body weight (ABW) has been reported to result in fluid administration that exceeds the amount actually required, thereby increasing the risk of fluid overload in obese patients [17]. Furthermore, when UV is indexed to ABW for the diagnosis of AKI, UV may be underestimated and AKI consequently overdiagnosed. In contrast, correction using ideal body weight (IBW) has been reported to provide greater validity in the diagnosis of AKI [18].

From a clinical perspective, lower UV on POD 1 in obese recipients should not be interpreted as a sign of graft dysfunction. Instead, it should prompt careful reassessment of volume status and graft perfusion. Our findings emphasize the importance of careful and individualized fluid titration to avoid both under-resuscitation, which may compromise graft perfusion, and over-resuscitation, which carries risks of fluid overload. Early physiological differences in UV should therefore be interpreted within the broader clinical context rather than as isolated indicators of poor outcomes.

This study has several limitations. First, its retrospective, single-center design limits generalizability. Second, UV and renal function were assessed only in the early postoperative period, and long-term graft outcomes and overall survival were not evaluated, because of the discrepancy in follow-up duration between the two groups. Finally, detailed hemodynamic parameters and biomarkers related to adipocytokines were not available, preventing direct mechanistic confirmation. Future prospective studies are warranted to further clarify the physiological mechanisms underlying these findings. 

## Conclusions

The Og undergoing LDKT demonstrated significantly lower UV on POD 1 and a slower initial recovery of graft function, as reflected by delayed creatinine reduction and eGFR improvement. However, these physiological differences did not intend a higher incidence of major postoperative complications or prolonged length of hospital stay. Although early UV may be lower in obese recipients, it suggests that careful and individualized perioperative fluid management is essential to maintain adequate graft perfusion.
